# Latency Reversing Agents: Kick and Kill of HTLV-1?

**DOI:** 10.3390/ijms22115545

**Published:** 2021-05-24

**Authors:** Annika P. Schnell, Stephan Kohrt, Andrea K. Thoma-Kress

**Affiliations:** FAU Junior Research Group “Retroviral Pathogenesis” and BMBF Junior Research Group in Infection Research “Milk Transmission of Viruses”, Institute of Clinical and Molecular Virology, Friedrich-Alexander-Universität Erlangen-Nürnberg (FAU), 91054 Erlangen, Germany; annika.schnell@fau.de (A.P.S.); stephan.kohrt@fau.de (S.K.)

**Keywords:** HTLV-1, ATLL, HIV, latency, kick and kill, shock and kill, Tax, latency reversing agents (LRA), HDAC-inhibitor (HDACi), P-TEFb

## Abstract

Human T-cell leukemia virus type 1 (HTLV-1), the cause of adult T-cell leukemia/lymphoma (ATLL), is a retrovirus, which integrates into the host genome and persistently infects CD4^+^ T-cells. Virus propagation is stimulated by (1) clonal expansion of infected cells and (2) de novo infection. Viral gene expression is induced by the transactivator protein Tax, which recruits host factors like positive transcription elongation factor b (P-TEFb) to the viral promoter. Since HTLV-1 gene expression is repressed in vivo by viral, cellular, and epigenetic mechanisms in late phases of infection, HTLV-1 avoids an efficient CD8^+^ cytotoxic T-cell (CTL) response directed against the immunodominant viral Tax antigen. Hence, therapeutic strategies using latency reversing agents (LRAs) sought to transiently activate viral gene expression and antigen presentation of Tax to enhance CTL responses towards HTLV-1, and thus, to expose the latent HTLV-1 reservoir to immune destruction. Here, we review strategies that aimed at enhancing Tax expression and Tax-specific CTL responses to interfere with HTLV-1 latency. Further, we provide an overview of LRAs including (1) histone deacetylase inhibitors (HDACi) and (2) activators of P-TEFb, that have mainly been studied in context of human immunodeficiency virus (HIV), but which may also be powerful in the context of HTLV-1.

## 1. Introduction

### 1.1. HTLV-1, a Persistent Human Tumorvirus

Human T-cell leukemia virus type 1 (HTLV-1) is a highly oncogenic retrovirus causing adult T-cell leukemia/lymphoma (ATLL) or inflammatory diseases like HTLV-1-associated myelopathy/tropical spastic paraparesis (HAM/TSP) in up to 10% of infected individuals [1,2,3,4,5]. Worldwide, at least 5–10 million people are infected with this yet neglected oncogenic retrovirus. Due to the restricted availability of reliable data, this is likely underestimated [6,7]. Asymptomatic carriers are mainly unaware of their infection and may pass the infection to other people since HTLV-1 infection is not part of sexual health screening in most countries [5]. The epidemiological distribution of HTLV-1 is unique. Endemic areas include, amongst others, Japan, the Caribbean, northern regions in South America, areas in intertropical Africa, and central Australia [6,8].

HTLV-1 poses as the etiological agent of malignant and inflammatory diseases, which develop after a clinical latency of years to decades. The aggressive T-cell malignancy ATLL and the inflammatory neurological conditions HTLV-1-associated HAM/TSP are of major concern [7]. Moreover, a plethora of other inflammatory conditions are known to arise from HTLV-1 infection, sometimes referred to as HTLV-1 associated inflammatory diseases (HAID) [9,10,11]. Data on the prevalence of these pathologies varies. Commonly, the lifetime risk for developing ATLL is estimated at around 3–5% for HTLV-1 infected people [12]. Roughly 2–3% of carriers develop HAM/TSP. There is only scarce data available on HAID prevalence [9,13]. The onset of these diseases is marked by a prolonged clinical and viral latency. Even though the conditions only develop in a low number of infected individuals, they are of clinical relevance as there are currently no effective therapies or vaccines available. As a result, the prognosis for patients suffering from ATLL is very poor. According to Shimoyama, ATLL can be classified into four clinical subtypes, with severance increasing from smoldering to chronic to lymphoma to acute. In aggressive types, the overall survival only amounts to approximately six to ten months [14,15].

Several comprehensive articles have summarized current treatment regimens for ATLL [15,16,17]. Options for ATLL depend on the subtype and vary between common chemotherapy regimens, treatment with interferon-α and antiretroviral agents, allogenic stem cell transplantation or may even include watchful waiting as an approach in the smoldering subtype [15,18]. One of the newer treatment options is Mogamulizumab, a humanized defucosylated monoclonal antibody targeting C–C chemokine receptor 4. It has been explored for the treatment of ATLL and HAM/TSP in clinical trials and is approved for the treatment of patients with relapsed or refractory CCR4^+^ ATLL in Japan [19,20]. However, HAM/TSP is still routinely treated only symptomatically with corticosteroids and other immunosuppressants or antiretrovirals [9]. Overall, there is a need to expand therapeutic options for HTLV-1-infected patients. Consequently, the discovery of efficient and potentially curative treatments is mandatory [21].

Owing to its retroviral nature, the HTLV-1 genome integrates into the host genome upon cell-to-cell spread and infection of CD4^+^ T cells. Interestingly, contrary to previous beliefs, the integration site is not random but targets a nonpalindromic DNA motif [22]. Virus propagation occurs either indirectly by mitotic and clonal expansion of infected cells or directly by de novo infection and transactivation of viral gene expression by the viral transactivator and oncoprotein Tax [7,23]. As a common characteristic of retroviruses, the integrated proviral HTLV-1 genome is flanked by long terminal repeats (LTRs) and encodes *gag*, *pol*, and *env* genes. Additionally, the δ-retrovirus genome possesses a unique pX region that features several open-reading frames encoding regulatory and accessory genes, including the oncogene and viral transactivator Tax, a central regulatory protein. Tax exerts not only a myriad of functions in the host, e.g., by stimulating the proliferation of infected host cells, but it is also a central player in viral replication [23]. Due to Tax-responsive promoter elements in both the 5′ LTR and the 3′ LTR, Tax activates plus (sense) and minus (antisense) strand transcription, respectively [7,24,25].

Regulation of viral gene expression and viral latency is subject to a meticulously fine-tuned balance of different viral gene products and cellular transcription factors. The mechanism through which Tax initiates HTLV-1 transcription is mediated via the cellular transcription factor cAMP response element binding protein (CREB) and has been reviewed previously [23,26]. Briefly, the viral LTRs feature three highly conserved 21 bp enhancer elements referred to as viral cAMP response elements (vCREs) or Tax-responsive elements (TREs). Interaction of Tax with phosphorylated CREB (pCREB) is required so that Tax can bind to the viral promoter via the TREs. Subsequently, the Tax/pCREB complex recruits the cellular histone acetyltransferase CREB binding protein (CBP) and p300 to the viral promoter. Acetylation of histones at the viral promoter results in nucleosomal remodeling and a more permissive chromatin state, overall favoring transcriptional activation [7,24,26,27,28]. Another determinant of transcription is the RNA polymerase II (RNA Pol II). Productive elongation by the RNA Pol II is facilitated by the positive transcription elongation factor b (P-TEFb), which releases the RNA Pol II from promoter proximal pausing. Tax is able to bind P-TEFb and enhance the activation and transcription of the viral promoter. Briefly, Tax not only affects the initiation of viral transcription but also recruits host cell factors like P-TEFb to the viral promoter to simulate transcription elongation [29,30].

### 1.2. The HTLV-1 Viral Reservoir

HTLV-1 preferentially infects CD4^+^ T-cells in vivo. However, it is also found to a minor extent in CD8^+^ T-cells, monocytes and dendritic cells [31,32,33]. HTLV-1-infected T-cells frequently have the phenotype of activated long-lived memory T-cells and are CD4^+^ CD25^+^ CCR4^+^ CADM1^+^ [34,35]. The latter marker cell adhesion molecule 1 (CADM1/TSLC1) was found as the best single marker of HTLV-1 infection, identifying HTLV-1 infected cells with greater sensitivity and specificity than CD25, CCR4, or ICAM-1 and showing that CADM1^+^ CD4^+^ T cells carried a median of 65% of proviral copies in peripheral blood [36]. However, for HTLV-1, the viral reservoir “outside” the peripheral blood is largely unknown. Due to difficulties accessing patient material, most studies focus on ex vivo analysis of peripheral blood, which only comprises a subfraction of all immune cells in vivo. Few studies with the closely related simian T-cell leukemia virus type 1 (STLV-1) suggested that hematopoietic cells in the bone marrow are infected. This could be confirmed by high-throughput sequencing of HTLV-1-infected humans, which identified identical integration sites in neutrophils, monocytes, B-cells, CD8^+^ T-cells, and CD4^+^ T-cells, which indicated that HTLV-1 infects hematopoietic stem cells (HSCs) in vivo. Thus, HSCs could not only contribute to viral spread but also to the formation of the latent reservoir [37].

Similar to the well-known retrovirus human immunodeficiency virus (HIV), the latent viral reservoir of HTLV-1 is the main obstacle to curing the infection. In the context of HIV, antiretroviral therapy (ART) has been used for years, however, ART cannot cure HIV due to the long-term persistence of latent HIV in resting and proliferating cells. HIV can reactivate and emerge from these latently-infected cells as soon as ART is ceased. Consequently, there is a need to eliminate latently infected cells and control HIV to achieve a cure of HIV or ART-free remission [38]. In HTLV-1-infected patients, infectious spread via cell-free virions is less active than in HIV-infected people during disease progression. Persistence of HTLV-1 in the host is predominantly mediated via mitotic spread and clonal proliferation [7,24]. However, it remains to be determined whether similar mechanisms may account for the treatment of HTLV-1-infected individuals after viral reactivation.

### 1.3. Immune Responses Targeting Tax

The Tax protein is not only required for activating viral transcription but also for the initiation of malignant transformation of infected cells, mostly CD4^+^ T-cells [39]. However, Tax expression is usually undetectable in freshly isolated peripheral blood mononuclear cells (PBMCs) from HTLV-1 infected subjects, but gets reactivated upon removal of CD8^+^ T-cells. Concomitant with that, a persistently activated cytotoxic T lymphocytes (CTLs) response is usually detectable, directed towards the immunodominant protein Tax even in latently infected individuals [7,24,40,41,42,43,44]. Interestingly, recent work identified that spontaneous bursts of Tax expression occur when PBMCs from HTLV-1 infected individuals are cultured ex vivo. A possible explanation for the sustained CTL response towards Tax is that Tax is also spontaneously expressed in vivo, though in intermittent bursts. Single-molecule RNA fluorescence in situ hybridization (smFISH) revealed that *tax* transcription occurs in infrequent bursts [24,45].

However, how is Tax expression repressed? In more than half of studied ATLL cases, plus-strand expression of HTLV-1 from the 5′ LTR is inactivated. This happens through genetic changes, such as mutations, insertions, or deletions or through epigenetic modifications, including DNA hypermethylation at the 5′ LTR, while the 3′ LTR remains unmethylated. In particular, North American ATLL patients display unique epigenetic alterations [46,47,48,49]. Moreover, viral gene expression is repressed in vivo not only by epigenetic mechanisms but also by viral and cellular proteins in late phases of infection, which has been reviewed earlier [24,50]. Thus, HTLV-1 avoids an efficient CTL response directed against the immunodominant viral Tax antigen. Consequently, new therapeutic strategies aim to transiently activate viral gene expression and antigen presentation of Tax to disturb the equilibrium in favor of an enhanced CTL response towards HTLV-1, thus exposing the latent HTLV-1 reservoir to immune destruction. In HTLV-1 infection, the proviral load is strongly correlated with the risk of developing HTLV-1-associated inflammatory diseases or ATLL. Strikingly, HTLV-1-specific CTLs can coexist with a high proviral load. Thus, efficient control of HTLV-1 in vivo depends on the CTL quality, in more detail, on CTL avidity and lytic efficiency [51]. The complex interplay between HTLV-1 and the immune response targeting HTLV-1 has been comprehensively reviewed elsewhere [7,52,53].

Interestingly, studies combining predictions with experimental validation in a large patient cohort (*n* = 432) showed that despite Tax being the immunodominant protein, CD8^+^ T-cells specific to the antisense protein HTLV-1 basic leucine zipper (HBZ) are the most effective CD8^+^ T-cells [54]. However, antigen presentation of HBZ is impaired, possibly due to nuclear retention of HBZ [55,56]. Thus, latency reversal may also be achieved by enhancing antigen presentation of HBZ, a topic that is very interesting, but not discussed within this review.

### 1.4. Kick and Kill, Shock and Kill, or Gene Activation Therapy to Achieve Latency Reversal

**Figure 1:**
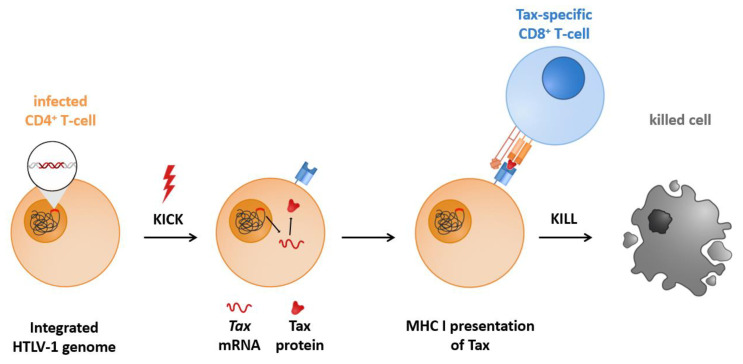
The Kick and Kill approach. The Kick and Kill approach presents a treatment option for HTLV-1 infection with the objective to eliminate the CD4^+^ T-cells latently infected with HTLV-1. The “KICK” aims to reactivate viral transcription. Consequently, the immunodominant protein Tax will be presented via MHCI on latently infected cells. CD8^+^ T-cells will mediate the “KILL” of this latent viral reservoir. Abbreviations: CD, cluster of differentiation; HTLV-1, human T-cell leukemia virus type 1; MHC I, major histocompatibility complex class I.

It would be desirable to prevent the outbreak of diseases in patients latently infected with HTLV-1. However, the central problem is eradicating the latent viral reservoir (see Section 1.2). Success in this field has been limited for HTLV-1. A therapeutic approach termed Kick and Kill, Shock and Kill, or gene activation therapy is to be described here (Figure 1). The goal is to eradicate latent reservoirs of infected T-cells. It might seem paradoxical to aim at activating viral gene expression, which can be equated with latency reversal. Nevertheless, transiently disturbing the tightly regulated viral latency attempts to force the virus out of hiding. Thus, the principle is based on exposing virus-positive cells via antigen-presentation to the host’s immune response and relies on a Tax-specific CTL response to eliminate latent viral reservoirs [57].

Lessons can be learned from the related retrovirus HIV-1. The virus, discovered in 1983, caused a pandemic and cost nearly 50 million lives. Research towards a cure for HIV-1 is still ongoing [58,59]. The primary obstacle is, similar to HTLV-1, the persistence of infected, quiescent but long-lived, and replication-competent CD4^+^ T-cells [58,60]. A possible cure for HIV-1 or any other latently persisting retrovirus would have to eradicate this latent viral reservoir. This Kick and Kill therapy, employing the immune response to kill infected cells, has been reviewed for HIV-1 infection prior [61,62,63]. Proof-of-concept was demonstrated in 2005, using the HDACi valproate and later also with the HDACi vorinostat. However, clinical results have been less promising in these studies and up to now [64,65,66]. The most intensively studied agents employed in HIV-1 latency reversal are bromodomain (BRD) inhibitors/bromodomain and extraterminal domain (BET) protein inhibitors (BETi), histone deacetylase inhibitors (HDACi), and methylation inhibitors. Among those, the prominent and diverse group of HDACi has been investigated the most [60,63].

### 1.5. Brief Categorization of Latency Reversing Agents (LRAs)

**Figure 2:**
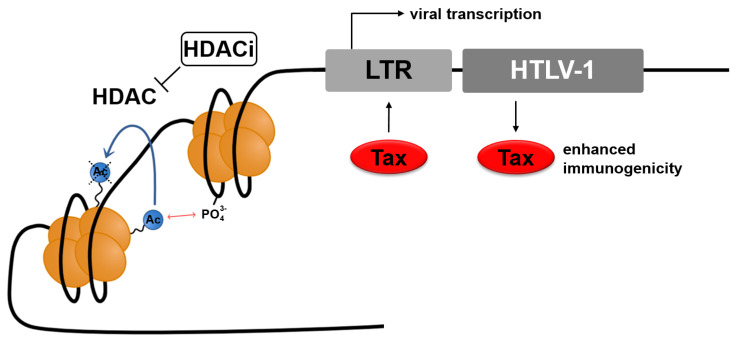
Mode of action of histone deacetylase inhibitors (HDACi) as latency reversing agents for HTLV-1. Epigenetic modifications govern transcription of the integrated HTLV-1 genome. Inhibition of histone deacetylases (HDACs) by histone deacetylase inhibitors (HDACi) raises acetylation of histone tails, thereby decreasing their affinity to chromatin. This results in a more permissive chromatin state, favoring transcriptional activation. Thus, the latent viral reservoir can be reactivated more easily. Abbreviations: HDAC, histone deacetylase; HDACi, histone deacetylase inhibitor; LTR, long terminal repeat; HTLV-1, Human T-cell leukemia virus type 1; Ac, acetylated lysine residue; ${\mathrm{PO}}_{4}^{3-}$ phosphate ion.

Compounds used to attempt latency reversal are called latency reversing agents (LRAs). Among them is the prominent group of HDACi. The widely accepted but probably oversimplified mechanism by which HDACi activate transcription is by inhibiting the deacetylation of histones (Figure 2). Acetylated lysine residues in histones inhibit their tight association with negatively charged DNA strands. As a result, less condensed chromatin is more likely to be transcribed [67]. There are several subgroups of HDACi, whose members differ in their pharmacological properties and their specificity towards the histone deacetylases (HDACs) they inhibit [68]. Hence, there is a distinction between pan-HDACi and class-selective inhibitors. It is not yet entirely elucidated, which histone deacetylases are located at the *HTLV-1* promoter. According to Lemasson, HDAC 1 and HDAC 2 are present at the 5′ LTR, and HDAC 3 is enriched at the 3′ LTR [69,70].

Another approach to reverse latency is the activation of P-TEFb. It comprises cyclin-dependent kinase 9 (CDK9) and cyclin T1, T2, or K and is crucial for the productive elongation of RNA-polymerase II. P-TEFb is found in an inactive, high molecular weight (HMW) complex with hexamethylene bisacetamide inducible protein 1 (HEXIM1) and 7SK snRNP and in an active low molecular weight (LMW) form, complexed with bromodomain-containing protein 4 (BRD4). Tax can competitively bind to P-TEFb, and activate HTLV-1 transcription more effectively than the other complexes [29]. Facilitating the binding of Tax to P-TEFb appears to be a profitable mechanism to stimulate transcription. By inhibiting different factors and thereby disrupting the HMW or LMW P-TEFb complex, it is possible to increase the amount of P-TEFb, which can bind Tax, resulting in HTLV-1 promotor activation [71,72].

Yet, systematic analyses and comparisons of compounds affecting HTLV-1 transcription are lacking. Moreover, the composition of the protein complexes guiding HTLV-1 gene expression and reactivation from latency is only partially understood. Thus, this review will provide an overview of LRAs focusing on (1) HDACi and (2) activators of P-TEFb, which have mainly been studied in the context of the related lentivirus HIV, but which may also be powerful in the context of HTLV-1. Finally, we will briefly summarize other strategies that aimed at enhancing viral reactivation, Tax expression, and Tax-specific CTL responses to interfere with HTLV-1 latency.

## 2. Manipulation of Histone Modification

### 2.1. Histone Modification

#### 2.1.1. Histone Modification: Histone Deacetylases (HDACs)

Epigenetic modifications of histone proteins strongly influence the transcription of genes. This justifies a closer look at histones. It has been established that the cell’s genetic information, the DNA, is complexed with core and linker histones. Core histones form octamer complexes, nucleosomes, consisting of two copies of each core histone: H2A, H2B, H3, and H4. Core histone protein domains comprise the histone-fold region, diverse extension, and histone tail [73,74]. The histones’ N-terminal tails are of particular interest, as they are exposed to post-translational modifications (PTM). Different enzymes can reversibly post-translationally modify these proteins. Acetylation, phosphorylation, methylation, ubiquitination, sumoylation, and ADP-ribosylation have been described. Modification of core histones influences their interaction with the nucleic acid. However, it can also become the basis for interaction with other proteins, referred to as “histone code” [75,76,77]. For example, acetylated lysine residues are recognized by bromodomains [78,79]. The most studied PTMs are histone acetylation and deacetylation. The responsible enzymes—histone acetyltransferases (HATs) and histone deacetylases (HDACs)—warrant an equilibrium of this particular histone modification [77]. Acetylation of lysine residues in histones has firstly been described in the 1960s [80,81]. Hydrolysis of these acetyl-lysine bonds is mediated by HDACs [82]. HDACs are categorized respectively to their homology to yeast HDACs into classes I, II, III, and IV. HDAC 1, HDAC 2, HDAC 3, and HDAC 8 belong to class I. They localize in the nucleus and are ubiquitously expressed in the human body. Class II HDACs are considerably larger than those of class I. Their localization also differs from class I, as they shuttle between the nucleus and cytoplasm, and their expression is tissue-specific [77]. They can be further divided into classes IIa (HDAC 4, 5, 7, 9) and IIb (HDAC 6, 10). HDAC 11 is the sole class IV HDAC and combines properties of class I and class II. Classes I, II, and IV share a Zn^2+^-dependency and are inhibited by HDACi. In contrast to that, class III HDACs or sirtuins (SIRT) are nicotinamide adenine dinucleotide (NAD^+^)-dependent and unaffected by HDACi [77,83,84,85].

#### 2.1.2. HDACs at the HTLV-1 Promoter

Regulation of HTLV-1 transcription is not yet fully understood. Research at the beginning of the millennium began to elucidate the role of HDACs in viral transcription. It was discovered that HDACs are distributed asymmetrically at the viral promoters of HTLV-1, the LTRs. HDAC 1 and HDAC 2 display higher accumulation at the 5′LTR, whereas HDAC 3 binds more strongly than HDAC 1 and HDAC 2 to the 3′LTR [69]. Interestingly, Tax and HDACs bind the viral promoter in a mutually exclusive manner. Thereto, Tax competes with HDACs to bind to the HTLV-1 promoter to activate transcription and to relieve the transcriptional repression mediated by HDACs. Apart from that, direct association of Tax and HDAC 1 has been demonstrated and suggested with HDAC 2 and HDAC 3 [69,86,87]. However, HDAC 1, HDAC 2, and HDAC 3 are all members of class I HDACs. Further studies are needed to characterize other HDACs and HDAC classes’ binding and regulatory patterns at the HTLV-1 promoter and to evaluate the differential expression of HDACs in HTLV-1 infected cells.

### 2.2. Histone Deacetylase Inhibitors (HDACi)

#### 2.2.1. HDACi as the Anticancer Drugs

Several histone deacetylase inhibitors are licensed as drugs or research subjects in various forms of cancer treatment [88]. The use of HDACi is a plausible approach, considering that epigenetic deregulation is a common feature in cancer development [89,90]. The rationale behind their use is to reactivate the transcription of genes silenced in malignantly transformed cells [89]. Commonly, growth arrest or apoptosis is reported after HDACi treatment of transformed cell lines [68,83,91]. This poses the question as to what their mode of action is.

#### 2.2.2. HDACi: Mode of Action

The inhibition of HDACs increases the acetylation of histones. Increased acetylation is associated with a more permissive, relaxed chromatin state, favoring transcription. Thus, the application of HDACi positively correlates with transcriptional activation. Surprisingly, after treatment with HDACi, only 0.5–20% of global gene expression is altered [92,93,94], indicating further regulatory implications. Furthermore, only a fraction of promoters affected by HDACi treatment displays an increase in transcription. Consequently, this quite simple explanation falls short of describing the pleiotropic changes observed after treatment with HDACi.

Next to histone deacetylation, other target proteins of HDACs also play a role in modulating gene expression. These include transcription factors, such as p53 and NF-κB, and proteins involved in cell cycle control, apoptosis, angiogenesis, and cell invasion [92,93,95].

Another modulation of transcription takes place via DNA methylation. Hypermethylation of promoters is linked to transcriptional silencing. Recruitment of HDACs to methylated regions acts in concert with DNA methylation to repress transcription. This mechanism is also affected by HDACi treatment, resulting in either increased or decreased methylation, suggesting complex regulation patterns, which are still poorly understood [92,96,97].

Additionally, histone acetylation can alter gene expression via the recruitment of epigenetic readers. Reader proteins recognize the “histone code” via specific protein domains. The domain identifying acetylated histones is the bromodomain (BRD) [93,98]. This review will elaborate further on bromodomain-containing proteins, such as BRD4, below in Section 3.2.1.

#### 2.2.3. HDACi: Immune Modulation

A common concern regarding the treatment with HDACi is their impact on the immune system. HDACi act via diverse mechanisms, which might also interfere in immune responses. It is well established that many HDACi, such as trichostatin A, vorinostat, and romidepsin, have anti-inflammatory effects by reducing proinflammatory cytokines’ production [92]. However, an exact immunomodulatory mechanism of action has not yet been conclusively proven. Another common observation is an increase of regulatory T-cells and their function upon treatment with HDACi [99]. Particular concern focuses on the antiviral immunity mediated by virus-specific CD8^+^ T-cells and how it might be affected by HDACi. Despite that, in vivo studies with the HDACi valproate have demonstrated a sustained cytotoxic T-cell response [100,101]. The HDACi chidamide has been shown to increase immune-cell mediated cytotoxicity, targeting tumor cells [102]. Still, it appears recommendable to monitor CD8^+^ T-cell lysis efficiency in further trials.

### 2.3. Classes of Histone Deacetylase Inhibitors (HDACi)

#### 2.3.1. HDACi: Overview of Classes

A pharmacophore model describing the typical structure of HDACi has been established. The general structure consists of a cap, binding to the surface of the HDAC and contributing to the ligand–receptor interaction. A hydrophobic linker domain connects the cap to a zinc-binding group (ZBG), posing as the functional group of the HDACi. The ZBG chelates and interacts with the zinc cation in the active site of HDACs [103,104,105].

In accordance with their ZBG, HDACi are divided into structural classes. Although the classes of HDACi are not always uniformly described, the most widely recognized are hydroxamates, benzamides, short-chain fatty acids (sometimes referred to as carboxylates in a broader context), and cyclic peptides. Other groups include epoxyketones, sometimes referred to as electrophilic ketones and hybrid molecules, and will not be a part of this review [82,94,103,106]. This review will focus on HDACi, which have been explored in the context of HTLV-1 infection or latency reversal or which seem to be promising candidates to the end of a Kill and Kill approach targeting HTLV-1 infected cells (Table 1).

#### 2.3.2. Hydroxamates

As the name suggests, hydroxamates possess hydroxamic acid as ZBG. Most HDACi are hydroxamates and chelate the active zinc ion of HDACs in a bidentate manner [94,103,104]. One of the first members of this group and one of the first discovered HDACi is trichostatin A (TSA). TSA is a natural product from *Streptomyces*. It displays potent anticancer and antiproliferative effects [103,105,123]. TSA exerts HDAC inhibition in a noncompetitive, reversible manner in a nanomolar range. Concerning HDAC selectivity, TSA is classified as pan-HDACi, defined as an inhibitor of class I, class II, and class IV HDAC activity. Still, TSA’s class I, class IIb HDACs and HDAC 5 inhibition is more potent than its HDAC 4, 7, 9, and 11 inhibition [107,123].

In the context of HTLV-1-infection, the impact of TSA on histone acetylation was demonstrated in SLB-1 cells, an HTLV-1-transformed T-cell line (Table 1). TSA was able to increase global histone acetylation of histone H3 and H4. Furthermore, TSA treatment achieved a 1.6-fold increase in viral RNA as measured by S1 nuclease protection assays. Additionally, MT-2 cells, HTLV-1 positive transformed T-cells, were treated with TSA. After treatment with TSA, transcripts from the 3′ LTR were also two- to three-fold enhanced [69,70,124]. Beyond that, TSA can enhance the viral expression of the bovine leukemia virus (BLV). BLV is a delta-retrovirus closely related to HTLV-1, which naturally infects cattle but is frequently used to experimentally infect sheep, which develop lymphomas [125]. The increase was assessed by employing luciferase assays using the BLV LTR and further demonstrated in PBMCs from BLV-infected sheep [109]. However, treatment of patients with TSA and clinical trials have not been pursued due to unfavorable side effects and TSA’s toxicity [103].

Another hydroxamate, which is already approved for the clinical treatment of cutaneous T cell lymphoma (CTCL), is suberoylanilide hydroxamic acid (SAHA, vorinostat, trade name Zolinza^®^; Table 1). In contrast to TSA, vorinostat is an irreversible HDACi. However, analogous to TSA, vorinostat is a pan-HDACi, although it displays higher IC50 values for HDAC 4, 5, and 9 [95,105,107]. The antiproliferative effect of vorinostat has also been demonstrated in HTLV-1 infected cells (MT-1, -2, -4, and HUT102), ranging in the micromolar range. Moreover, vorinostat induced cell cycle arrest and apoptosis. A possible implicated mechanism is the disruption of NF-κB signaling [112]. Furthermore, vorinostat can enhance Tax expression in MT-1-GFP^+^ reporter cells [110]. Research concerning HTLV-1 associated ATLL is up to now focused on the anticancer effects of vorinostat as opposed to its latency-reversing abilities [126] (NCT00005634, NCT01116154, and NCT00499811). As an anticancer agent, Vorinostat achieved a response rate of 29.7% in phase IIb trials in patients, displaying persistent, progressive, or treatment refractory cutaneous T-cell lymphoma [127]. Encouragingly, the side effect profile of vorinostat is manageable [126,127,128]. Trials in patients concerning the Kick and Kill approach have not yet focused on HTLV-1. However, studies with patients suffering from HIV-1 infection were able to show that vorinostat is moderately potent at latency reversal of this retrovirus [64,108,129,130]. Despite these promising results, concerns remain about the limitations of drugs such as vorinostat. Even though vorinostat could raise HIV transcription, this effect does not necessarily translate to viral protein expression and virion production, potentially due to a post-transcriptional block [131,132].

Belinostat (PXD-101, trade name Beleodaq) is another HDACi already approved to treat peripheral T-cell lymphoma (PTCL). It has its pan-HDACi activity in common with the above-mentioned HDACi and is active at a nanomolar to low micromolar range. Belinostat displays low toxicity in patients, and side effects are mostly manageable [84,95,133,134,135,136]. Belinostat is potent at inhibiting cell proliferation [137] and has successfully stimulated cell lines and primary CD4^+^ T-cells, latently infected with HIV-1, to express virus [111] (Table 1). However, PubMed searches with the keywords “belinostat”/“PXD”/“Beleodaq” and “htlv” or the related retroviruses BLV “blv” and STLV “stlv” retrieved no results (on 11 May 2021). Clinical research of ATLL is also more focused on the anticancer properties of belinostat than on latency reversal of HTLV-1 (NCT00354185, NCT02737046).

Another member of the hydroxamic acid-derived HDACi is panobinostat (LBH589, trade name Farydak), which is approved for the treatment of patients suffering from multiple myeloma. Panobinostat is a close analog to LAQ824 [85,105,138]. Like the HDACi mentioned earlier, it acts as pan-HDACi, active against class I, class II, and class IV HDACs. The potency of panobinostat lies in the nM range. However, it requires higher IC50 values for the inhibition of HDAC 4, 7, and 8. In general, panobinostat is well tolerated in the clinical setting [139,140,141]. Panobinostat has shown similar effects to vorinostat in HTLV-1 infected cell lines, namely inhibition of proliferation and enhancement of Tax expression (Table 1). Yet, it is notable that these effects already occur at a nanomolar concentration due to its greater potency in comparison to vorinostat [110,112]. Similar to vorinostat, blockade of NF-κB signaling is implicated in panobinostat’s mode of action [112]. Moreover, it has been shown that the apoptosis of ATLL-derived cell lines is induced via a RAIDD-caspase-2 pathway [142]. Furthermore, studies in patients, cell lines, and primary cells latently infected with the related retrovirus HIV-1 demonstrate that panobinostat is significantly more potent in inducing viral production than other HDACi, such as vorinostat and valproate. It is worth noting that panobinostat was already active at concentrations below standard dosing [111,141]. There are currently several ongoing clinical trials, which also target ATLL, but similar to trials with vorinostat, they mostly focus on the agents’ anticancer properties (NCT01261247, NCT00962507, NCT00918333, and NCT00699296), but not on latency reversal or viral reactivation.

#### 2.3.3. Benzamides

Benzamide derivatives also belong to the group of HDACi. Their ability to inhibit HDACs relies on an amino group as ZBG to chelate the zinc ion of the HDACs [103,105]. They are, in general, less potent than hydroxamates but possess greater class I selectivity [82,94,104]. Their lead compound is entinostat (MS-275). It inhibits HDAC 1, 2, and 3, representing a subset of class I HDACs, in a micromolar range. Data on inhibition of HDAC 8 and 9 is inconclusive [95,103,105,143]. In clinical trials, entinostat has been well-tolerated but is not yet approved for treatment. Its antitumor activity has been demonstrated in vitro and in vivo [94,103,143]. In HTLV-1 infection, entinostat has been shown to inhibit proliferation of the HTLV-1 infected cell lines (MT-1, -2, and -4) and primary cells (Table 1). Entinostat also blocked NF-κB signaling [112]. In cell lines (primary CD4^+^ T-cells, ACH2, and J-lat 6.3), latently infected with HIV-1, it has been demonstrated that entinostat can achieve robust viral replication. In contrast to that, the efficacy of panobinostat differed more between different cell models. Due to the selective class I inhibition of HDACs by entinostat, fewer side effects during treatment might be expected [113].

Chidamide (HBI-8000, trade name Epidaza) is another member of the Benzamide class of HDACi. It inhibits the class I HDAC 1, 2, and 3 and further the class IIb HDAC 10 in a low nM range. Class I HDAC 8 and class IV HDAC 11 are inhibited at a higher IC50. Chidamide is already approved for the treatment of PTCL in China [84,102]. In ATLL derived cell lines and primary cells from ATLL patients, chidamide has proapoptotic effects (Table 1). Interestingly, this effect is associated with the activation of Bim, a proapoptotic molecule, downregulated by the HTLV-1 Tax protein [144]. A phase 2 trial has evaluated the safety and efficacy of chidamide in patients suffering from ATLL (NCT02955589). In the context of HIV-1 infection, chidamide can reactivate the latent viral reservoir through NF-κB signaling. It is a safe treatment option and can induce intermittent viremia in HIV-1 positive patients and, beyond that, a modest decrease in viral DNA [114,115]. Based on chidamide’s and entinostat’s ability to reactivate HIV-1 from latency, these agents should be further evaluated in the context of HTLV-1 latency reversal.

#### 2.3.4. Short-Chain Fatty Acids

In general, the zinc ion binding capabilities of short-chain fatty acids rely on carboxylate groups and are weaker than those of the other HDACi groups. Thus, their IC50 values lie in a millimolar range [82,105]. Butyrates, such as sodium butyrate and phenylbutyrate, are derivates of butanoic acid. They have been explored for the treatment of malignancies and show minimal side effects [135,145]. Butyrate is part of a regular diet and a metabolite of fermentation in the gut [82,146]. Class I and class IIa HDACs are inhibited by butyrate [116].

The ability of sodium butyrate to increase histone acetylation at the HTLV-1 promoter was demonstrated in the HTLV-1 infected T-cell line SLB-1 (Table 1). Moreover, treatment of the cells with sodium butyrate resulted in a 2.4-fold increase in viral RNA [70]. Furthermore, sodium butyrate could also robustly induce Tax protein expression in the HTLV-1 infected T-cell lines 1996, 1657, HUT 102, FS, and A212 [117].

Valproic acid/valproate is an anticonvulsant used for the treatment of epilepsy. Due to the long time, it has been in use, its pharmacological properties are established, and it is known to be well-tolerated [94]. Additionally, it has been discovered that valproate also acts as an HDACi [147,148]. Similar to butyrates, millimolar concentrations are required for HDAC inhibition, inhibiting class I and class IIa HDACs [105,118]. Proof-of-concept for the Kick and Kill approach with valproate to eliminate latently infected cells in patients with HIV-1 infection was achieved in 2005. In patients treated with valproate, the population of infected resting CD4^+^ T-cells was reduced [66]. Moreover, it was shown that valproate treatment is proapoptotic and can increase transcription from the BLV-promoter [109,119] (Table 1). In BLV-infected sheep, valproate increased and thereafter decreased the viral load, which strongly suggests and supports the proposed Kick and Kill model [119]. However, the occurrence of chemoresistance to valproate has also been described. As a consequence, a modified HDACi, ES8, which possesses a hydroxamic acid as functional group, was tested in four BLV-infected sheep. In one out of these sheep, ES8 strongly decreased the proviral load and nearly cleared leukemia [149]. STLV-1 infection poses another good animal model for HTLV-1 infection. In baboons infected with STLV-1, valproate transiently raised the proviral load. The combination of valproate and the antiretroviral drug azidothymidine prevented this increase and achieved an overall decrease in proviral load. This occurrence is likely due to azidothymidine, preventing new cells’ infection and, additionally, the host’s immune response [101]. Valproate can also stimulate transcription from the *HTLV-1* promoter as shown in HeLa cells and Jurkat T-cells transiently transfected with HTLV-1 LTR-luciferase plasmids. Interestingly, valproate modulates Tax expression from the 5′ LTR and inversely impacts HBZ expression from the 3′ LTR. The increase of histone H3 acetylation further underlines the mode of action of valproate as HDACi. Moreover, valproate was found to induce apoptosis in lymphocytes from HAM/TSP patients, but also in lymphocytes from healthy persons and non-T-cells. The potency of valproate has also been demonstrated in vivo. In HAM/TSP patients, valproate transiently increases the proviral load but thereafter results in an overall decrease of the proviral load [57,120]. Concerns have been raised about the safety of treatment with valproate and, in particular, the immunomodulatory effects of HDACi (see Section 2.2.3). A 2-year trial demonstrated the safety of long-term treatment with valproate, and it has shown that the CD8^+^ T-cell function is not impaired. However, this trial did not report an overall decrease in the proviral load of HAM/TSP patients [100]. Thus, it should be determined whether HDACi more potent than valproate could achieve a more robust decrease in the proviral load of patients infected with HTLV-1.

#### 2.3.5. Cyclic Peptides

Cyclic peptides are the class of HDACi with the most complex cap groups. Their cap groups possess macrocycles, which lend them the ability for distinct interactions with the surface of the HDACs. These interactions pose the basis for selective inhibition [82,104]. Furthermore, they consist of very potent inhibitors, similar to the hydroxamates. Thus, they are active in a nanomolar range. Cyclic peptides are divided into two subclasses: containing or lacking an epoxyketone group. HDACi with an epoxyketone act irreversibly, and without an epoxyketone, they act reversibly on the HDACs [82,94].

The cyclic peptide romidepsin (FK228, depsipeptide, trade name Istodax) is a natural product from *Chromobacterium violaceum*. Romidepsin is already approved for the treatment of CTCL and is well-tolerated [150,151,152]. Romidepsin does not contain an epoxyketone group. Thus, it acts reversibly. Furthermore, romidepsin functions as a pro-drug. It contains a disulfide bond, which has to be intracellularly reduced by glutathione reductase to a thiol group. The thiol group is a potent ZBG to chelate the zinc ion at the active site of the HDACs [94,104]. Romidepsin inhibits the class I HDAC 1, 2, and 3 in a low nanomolar range. HDACs of class II are only inhibited at a higher concentration [84,104,153,154].

The growth-inhibiting effect of romidepsin was demonstrated in the context of HTLV-1 infection (Table 1). In HTLV-1 positive cell lines (HUT 102, CaGT, MT-2, MT-1, and MJ), romidepsin inhibited proliferation and induced apoptosis [121,152,155]. Increased histone H3 acetylation underlines its mode of action. Romidepsin also enhances Tax protein expression in the HTLV-1 infected T-cell lines HUT 102 and MT-2 [121]. The antitumor effect of romidepsin for ATLL has been demonstrated in murine models of ATLL. Romidepsin inhibited tumor growth and prolonged survival of the animals [121,155]. A clinical trial is ongoing (NCT04639843). Overall, this suggests that romidepsin is a promising candidate for the Kick and Kill approach in HTLV-1 infection.

To conclude, the use of HDACi in the latency reversal of HTLV-1 to the end of a Kick and Kill method presents a promising approach. Further systematic research and analysis of HDACi is an encouraging step to improve the treatment of HTLV-1 infection.

## 3. Agents for Targeting P-TEFb Complexes

### 3.1. Regulation of the RNA Polymerase II

The regulation of gene expression is a highly orchestrated process in mammals. The main role in this orchestra is played by the RNA Pol II, which forms the basis for all cellular activities by the transcription of DNA into precursors of messenger RNA [156,157]. Transcription by RNA Pol II is conserved in eukaryotes and is defined by sequential stages of initiation, elongation, and termination [158]. The activity of RNA Pol II and, therefore, the progression through these steps of transcription is a highly controlled process with multiple transcription factors involved [159,160,161]. A unique selling point of RNA Pol II is the carboxy terminal domain (CTD) of its subunit Rpb1. The CTD plays a central regulatory role since it provides a multifunctional interaction platform for factors regulating RNA Pol II. In humans, the interaction platform consists of 52 repeats of the conserved consensus sequence YSPTSPS [162,163]. The site and the status of phosphorylation of this heptapeptide repeat are important for the regulation of RNA Pol II activity. The RNA Pol II CTD is hypophosphorylated during the recruitment to the gene and gets phosphorylated at serine 5 (Ser5) by the serine/threonine kinase CDK7, which is associated with the transcription initiation factor II H (TFIIH), and at serine 2 (Ser2) by CDK9, a component of P-TEFb [164,165]. The phosphorylation of the CTD at Ser2 (Ser2P) is an indication for long range-elongation, whereas the phosphorylation at Ser5 (Ser5P) without Ser2 phosphorylation is mostly seen in the initiation phase of transcription [166,167]. Interestingly, there is a clear shift of high Ser5P to high Ser2P in line with transcription elongation levels, resulting in a decrease of Ser5P and an increase of Ser2P [166,168,169,170,171].

P-TEFb is a heterodimer consisting of the catalytic subunit CDK9 and either cyclin T1 or cyclin T2 as a regulatory subunit [172,173]. In the absence of P-TEFb, RNA synthesis via the RNA Pol II elongation machinery pauses 30–40 nucleotides downstream of the transcription start site in most human genes in the absence of P-TEFb [174,175,176]. While this promotor proximal pausing (PPP) stabilized by the negative elongation factor (NELF) and the DRB-sensitivity inducing factor (DSIF), transcription can be reinitiated by phosphorylation of Ser2-CTD, of SPT5 (a subunit of DSIF), and of RD (a subunit of NELF) by P-TEFb [161,177,178,179,180,181,182,183,184].

The RNA synthesis by RNA Pol II is not a ride along non-stop process since the Ser5P-RNA Pol II elongation machinery pauses 30–40 nucleotides downstream of the transcription start site in most human genes in the absence of P-TEFb [174,175,176]. The negative elongation factor (NELF) and the DRB-sensitivity inducing factor (DSIF) are stabilizing this promotor proximal pausing (PPP). The release from this PPP is modulated by the phosphorylation of Ser2-CTD, of SPT5 (a subunit of DSIF), and of RD (a subunit of NELF) by P-TEFb [161,177,178,179,180,181,182,183,184].

### 3.2. Different P-TEFb Modulating Agents

**Figure 3:**
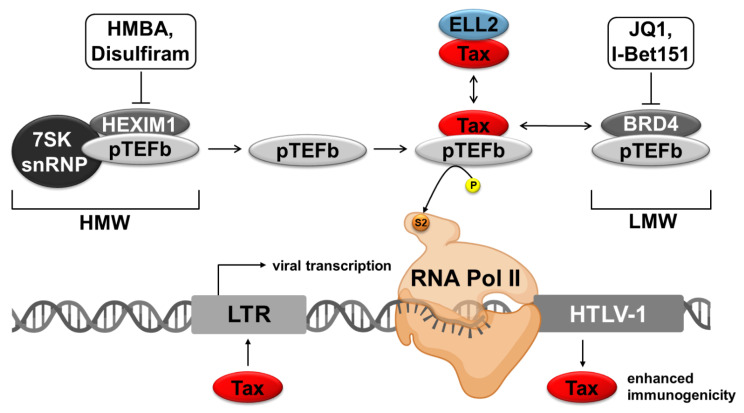
Activators of pTEFb as latency-reversing agents for HTLV-1. The cellular function of the positive transcription elongation factor b (P-TEFb) is to release the RNA Pol II from promoter proximal pausing by phosphorylating Ser2 in the carboxyterminal domain. The level of activity of P-TEFb is governed by its existence in complexes with different cellular proteins. In the inactive high molecular weight complex (HMW), P-TEFb interacts with HEXIM1 and 7SK snRNP; in the low molecular weight complex (LMW), P-TEFb complexes with BRD4, thus, activating transcription. Targeting these complexes to achieve the release of P-TEFb facilitates the binding of P-TEFb to the viral transactivator Tax. The complex of pTEFb and Tax could activate HTLV-1 transcription most effectively, resulting in enhanced Tax protein expression. Additionally, complexes between the P-TEFb-related transcription elongation factor ELL2 and Tax exist. Abbreviations: P-TEFb, positive transcription elongation factor b; 7SK snRNP, 7SK small nuclear ribonucleoprotein; HEXIM1, hexamethylene bisacetamide inducible protein 1; HMBA, hexamethylene bisacetamide; BRD4, bromodomain-containing protein 4; LTR, long terminal repeat; HTLV-1, human T-cell leukemia virus type 1.

P-TEFb activity is controlled by the interaction with different proteins. The kinase-active form of P-TEFb is described as a LMW complex and predominantly binds the BRD4, subunits of the super elongation complex (SEC), or other DNA bound activators [29,162,185,186] (Figure 3). In contrast, the larger inactive P-TEFb complex with a HMW is associated with the 7SK snRNP and the HEXIM1 protein [187,188]. The ratio between HMW and LMW is highly modulated [173,189,190].

In the context of HTLV-1, Tax competitively binds to the cyclin T1 subunit of P-TEFb to activate viral transcription [29]. Thus, strategies to interfere with the formation of the LMW or HMW to provide “free” P-TEFb for complex formation with Tax could enhance viral transcription. In HIV-1 infection, a so-called super elongation complex is recruited to the viral promoter to foster transcription elongation. This SEC is recruited by Tat to the viral promoter and composed, amongst others, of the scaffold protein AFF4, P-TEFb, and ELL2, which is another transcription elongation factor [186,191]. Interestingly, ELL2 is the stoichiometrically limiting component of this SEC. For HTLV-1, it is unknown whether a comparable SEC exists. However, ELL2 is strongly and specifically upregulated in chronically HTLV-1 infected cells, enhances Tax-mediated viral transactivation of the *HTLV-1* promoter, and ELL2 and Tax are part of a common protein complex [192]. Yet, it is unclear whether Tax:ELL2 complexes and Tax:P-TEFb complexes are part of a common complex and how this complex impacts viral reactivation.

#### 3.2.1. Agents Interfering with the LMW-Complex

BRD4 is ubiquitously expressed in the nucleus and can interact with acetylated histones H3 and H4 [193,194,195]. Moreover, as a member of the BET family, BRD4 is characterized by its two conserved tandem bromodomains, which are crucial for the histone interaction and an extraterminal domain [196]. As the main component of the LMW-complex, BRD4 recruits P-TEFb to the promotor so that the kinase CDK9 can mediate transcriptional elongation by phosphorylation of the CTD of the RNA Pol II. It could be shown that BRD4 transactivates not only cellular but also viral promoters [189]. In the context of HTLV-1, Tax is a viral competitor of BRD4 for the interaction with the P-TEFb subunit cyclin T1, another main component of the LMW-complex. Besides this, BRD4 also inhibits Tax-dependent transactivation of the HTLV-1 promotor dose-dependently [29,197]. Together, these findings strongly underline the potential of BRD4 inhibitors or P-TEFb releasing agents as a possibility to reactivate HTLV-1 from latency. Agents inhibiting bromodomains and extraterminal domains are commonly known as BETi [198]. Currently, manifold BRD4 inhibitors already exist. However, they have been predominantly used in the context of HIV-1 rather than HTLV-1.

One of the most famous BRD4 inhibitors is the thienotriazolodiazepine compound JQ1 (Table 2; Figure 3). The Mitsubishi Tanabe Corporation described thienodiazepine analogs as antitumor compounds inhibiting the binding of acetylated histones with bromodomain containing (BET) proteins, which laid the foundation for compounds like JQ1 [199] WO/2009/084693). Soon after, JQ1 was described as effective in preclinical animal trials since JQ1 treatment resulted in tumor regression and improved survival in murine models of the so-called nuclear protein in testis (NUT) midline carcinoma (NMC) [78]. To further characterize the functionality, JQ1 stereoisomers were analyzed by fluorescence recovery after photo bleaching (FRAP) experiments and cocrystal structure analysis, which revealed that only (+)JQ1 is biochemically active [78]. The potential to interfere with the interaction of acetylated histones and BET-proteins makes JQ1 therapeutically useful in various diseases. The broad effect of JQ1 on different cancers, including hematological malignancies and solid tumors, has been confirmed, and the antitumor activity can be explained by the decreasing effect of JQ1 on the expression of cell proliferating genes [78,200,201,202]. Moreover, JQ1 is not only useful in the context of cancer but also tested in viral infections. For HIV-1, it could be shown that JQ1 promotes HIV-1 transcriptional reactivation with minimal cytotoxicity while suppressing T-cell activation and inducing histone modification genes and cyclin T1 [203]. Zhu et al. could extend these findings by showing that JQ1 increases proviral transcriptional elongation if used in combination with the protein kinase C activator prostratin or phytohemagglutinin (PHA) [204]. Interestingly, the FDA-approved drug ingenol-3-angelate (PEP005) can act synergistically with JQ1. The treatment of U1 cells, an HIV latency model, with PEP005 led to a 25-fold induction of viral transcription, whereas the combination of PEP005 with JQ1 enhanced the induction 250-fold compared to controls. Additionally, in ex vivo experiments with CD4^+^ T-cells from patients receiving ART, the number of full-length HIV-transcripts increased at least 2-fold in five out of seven patients 6 h after treatment with PEP005 [205]. Furthermore, JQ1 induced apoptosis and cell cycle arrest in the HTLV-1 infected cell lines Rat-1-Tax, MT-4, C8166, and SLB1 [206]. Notably, JQ1 raised Tax expression in the HTLV-1 infected MT-1-GFP^+^ reporter cells. This enhancing effect on HTLV-1 transcription could be boosted by administering JQ1 in combination with phorbol 12-myristate 13-acetate (PMA, 12-*O*-tetradecanoylphorbol-13-acetate, TPA) and ionomycin [110] (see Section 4.2).

Another study not only showed reactivation of HIV from latency upon JQ1 treatment but also upon I-BET-151 treatment, another BRD4-inhibitor (Table 2; Figure 3). They could show that both BET inhibitors reactivate HIV from latency in a Tat-independent mechanism in latently HIV l infected polyclonal Jurkat cell populations and primary T-cell models [72]. The compound I-BET-151 could reactivate HIV-1 in cells in vivo under a combinational antiretroviral therapy (cART). Moreover, experiments with humanized mice under cART treated with I-BET-151 further showed that HIV-1 genes were preferentially CDK9- and CDK2-dependently expressed in monocytic cells, but not in CD4^+^ T-cells whereas the underlying mechanism for this differential effect of I-BET-151 is unknown so far. [207].

A novel thienotriazolodiazepine compound and BETi/BRD4-inhibitor OTX-015 (Birabresib), with less cytotoxic effects, has already entered phase 1b clinical trials (NCT01713582; Table 2). Even the highest concentration (5 µM) of OTX-015 was responsible for only less than 20% of drug-specific cell death in CD4^+^ and CD8^+^ T-cells [219]. In C11 cells, OTX-015 could dose-dependently stimulate HIV-LTR transcription with a nearly twice as low EC50 value as compared to JQ1. OTX-015 could dose-dependently stimulate HIV-LTR transcription with a nearly twice lower EC50 value than JQ1 in C11 cells. Further, OTX-015 could reactivate HIV from latency with a more than four times lower EC50 value than JQ1 in J.Lat clone A10.6 cells. OTX-015 could even induce latent HIV-1 expression ex vivo in primary CD4^+^ T-cells receiving ART. Additionally, the treatment with OTX-015 corresponded with a nearly 10-fold increased RNA Pol II CTD Ser2 phosphorylation, and the level of HIV-1 promotor-bound CDK9 was increased by more than 5-fold. Concluding, the HIV-1 reactivation by OTX-015 in all of these samples is P-TEFb dependent [209].

A second generation 3,5-dimethylisoxazole BETi derived from an Imidazo [1,2-a] pyrazine scaffold, named UMB-136, is another promising compound for HIV-1 latency reversal. Hunag et al. screened 37 UMB-32 derivatives that demonstrated inhibitory effects on BRD4, combined with easier synthesis than JQ1. Based on UMB-32, the analog UMB-136 was evaluated as the most promising BETi. Compared to the other analogs, UMB-136 contains a cyclohexane group and the lowest binding energy for the binding with BRD4, even lower than JQ1, underlining the great binding potential of UMB-136 to BRD4. In addition, UMB-136 binds endogenous BRD4 and leads to an enhanced Tat:P-TEFb interaction. The treatment with UMB-136 reversed HIV-1 latency in most HIV-latency models, including the cell lines J-Lat 6.3, 8.4, 9.2, 10.4, and THP89-GFP and in naïve CD4^+^ T-cells analyzed, whereas the treatment with JQ1 alone showed no effect compared to the controls [210]. Since the combination of different latency reversal agents has shown to be a promising approach [198,220], synergistic effects of UMB-136 with other LRAs were compared to JQ1 synergism. In this setting, treatment with UMB-136 revealed to achieve greater effects compared to JQ1. For JQ1, a tremendous synergism has been reported for double treatment with the PKC agonist bryostatin-1 [198,220]. A highly synergistic effect on latency reversal was also observed upon treatment of cells with UMB-136 together with bryostatin-1, which was again greater than the effects observed upon treatment with JQ1 and bryostatin-1 [210].

Summed up, a considerable repertoire of BRD4 inhibitors already exists, which was analyzed for latency reversal of HIV-1. Further, a combination of different compound classes revealed promising results, too. However, neither the novel inhibitors nor the combinatorial approaches have been tested for HTLV-1.

#### 3.2.2. Agents Interfering with the HMW

Besides agents interfering with the LMW, other compounds releasing P-TEFb independent of BRD4 exist. The binding of the negative regulator HEXIM1 and of the small nuclear RNA species (7SK snRNP) to P-TEFb leads to an inhibition of the kinase activity of pTEFb, which makes HEXIM1 and 7SK snRNP negative regulators of P-TEFb [221] (Figure 3). The mechanistic background of this HMW complex formation is defined by the conformational change of a HEXIM1 homodimer induced by 7SK snRNA binding, which finally facilitates them to bind to P-TEFb [173,212]. The ratio between HMW and LMW-complexes in the cell is tightly controlled [173,189,190]. Stress factors, such as actinomycin D, 5,6-dichloro-1-β-d-ribofuranosylbenzimidazole (DRB), UV-radiation, or inhibition of transcription lead to a shift from HMW to LMW-complexes, which in turn leads to enhanced transcription and translation [187,222,223]. In the context of HTLV-1, it could be shown that CDK9 and Tax are essential for viral transactivation and that Tax competes with key components of the LMW and HMW-complexes for P-TEFb binding [29,224]. Since the immunodominant protein Tax is essential for viral transactivation but is not expressed during latency, P-TEFb releasing compounds interfering with the HMW could give start-up support (“kick”) for the viral transcription, allowing the immune system to destroy the latent reservoir (“kill”).

In contrast to the frequently neglected virus HTLV-1, different compounds interfering with HMW complex formation have already been tested in the context of HIV-1. One of the oldest known compounds is the hybrid polar compound HMBA [225,226] (Table 2; Figure 3). In chronically infected T-cell lines, HMBA has demonstrated its huge potential in reactivating HIV-1 from latency [226,227]. Furthermore, HMBA can strongly increase the expression of the LMW component HEXIM1 [228,229]. Contreras et al. have shed more light on the mechanistic background of how HMBA induces viral gene expression. They reported that HMBA activates the phosphatidylinositol 3-kinase (PI3K)/Akt pathway by interfering with PI3K, leading to the dissociation of the HMW-complex, which in turn allows the recruitment of P-TEFb to the viral promotor [71]. The induction of viral production could be shown in chronically infected latency model cell lines (U1, ACH-2, and JΔK).

Another promising hybrid polar compound of the second generation is suberoylanilide hydroxamic acid (vorinostat/SAHA; Table 2), which is not only an HDACi (see Section 2.3.2), but can also activate the PI3K/Akt pathway, thus, affecting viral reactivation by the transient release of free P-TEFb from 7SK snRNP comparable to HMBA [214,226,230,231,232]. Interestingly, in resting CD4^+^ T-cells vorinostat treatment leads to an increased CDK9 T-loop (Thr-186) phosphorylation, required for P-TEFb activity [213,214]. In glycerol gradient experiments, it could be shown that cyclin T1 and CDK9 moved from higher (HMW) to lower (LMW) glycerol fractions at 90 min after the addition of 5 µM vorinostat [169].

Disulfiram [bis(diethylthiocarbamoyl) disulfide] is FDA approved and was initially used to treat alcoholism [233,234]. In Bcl-2 transduced CD4^+^ T-cells, disulfiram leads to a reactivation of latent HIV (Table 2; Figure 3). Moreover, disulfiram converts to di-ethyl-di-thio-carbamic acid (DDTC) in vivo, and none of both compounds showed significant toxicity if administered in concentrations lower than 10 µM [217]. Mechanistically, disulfiram interferes with the PI3K/Akt pathway by depleting phosphatase and tensin homolog deleted on chromosome 10 (PTEN), which results further downstream in phosphorylation of HEXIM, the release of P-TEFb, and transactivation of the viral promotor [216]. Interestingly, disulfiram was administered in a dose-escalation study to HIV-infected adults receiving suppressive antiretroviral therapy, where disulfiram treatment resulted in an increase of unspliced HIV RNA and was well-tolerated [215].

Another potential compound for retroviral latency reversal is trichostatin a (TSA; Table 2). Luciferase assays with transfected HeLa cells revealed a 30-fold induction of the HEXIM1 promotor after 48 h of treatment with TSA [235]. Besides transcriptional activation of HEXIM1, TSA can also release P-TEFb from the HMW complex [211,230]. Moreover, TSA was analyzed in combination with nicotinamide (NA). By using a reporter plasmid in 293T cells, it could be shown that the treatment of TSA/NA leads to a time-dependent reversible release of HEXIM1 from cyclin T1, and an ex vivo kinase assay revealed enhanced CDK9 kinase activity [218]. The impact of TSA on HTLV-1 transcription is described in Section 2.3.2 and Table 1 since TSA is also known as pan-HDACi.

The last P-TEFb releasing agent discussed here is an analog of cytidine named 5-Azacytidine (AzaC; Table 2). As a nucleoside analog, AzaC interferes with cell proliferation on a cellular level by being incorporated into DNA [236], but it has also shown potential in HIV transcription activation [237,238,239]. The potential of AzaC as a P-TEFb releasing agent was shown by a so-called V-PAC assay, in which the activation of P-TEFb is visualized via fluorescence complementation and by a glycerol gradient assay in HeLa cells [185]. In the context of HTLV-1, the viral reactivation from latently-infected cell lines MT-1 and TL-Om1 has been observed upon treatment with AzaC [47].

In conclusion, besides the use of HDACi, P-TEFb releasing agents represent a potent alternative for reversing the latency of HTLV-1. These compounds offer the possibility to release P-TEFb from the HMW complex or the LMW complex. Since released P-TEFb could complex with Tax to promote viral transcription, it is reasonable to study the potential of P-TEFb-modulating agents either individually or in combination with other LRAs to reactivate HTLV-1 from latency.

## 4. Other Strategies to Reactivate HTLV-1 from Latency

### 4.1. Classification of Other Stimuli

Thus far, HDACi and activators of P-TEFb have been outlined as LRAs. Beyond these agents, other stimuli can also activate HTLV-1 transcription. These include mitogens, sirtuin inhibitors, and extracellular factors. Moreover, also metabolic circumstances affect HTLV-1 latency. These regulators of HTLV-1 latency will be briefly reviewed in this section.

### 4.2. Mitogens

T-cell activating mitogens are known for their potency to enhance Tax protein levels in HTLV-1 infected T-cell lines (Table 3). Among them are plant lectins and phorbol esters [117]. Phytohemagglutinin-P (PHA) is a plant lectin that exerts a mitogenic stimulus on T-cells. It is also used to stimulate PBMCs from HTLV-1 infected patients [240]. In the HTLV-1 infected T-cell line FS, PHA can induce Tax protein expression [117].

A commonly used phorbol ester to stimulate retroviral LTRs, among them the HTLV-1 LTR, is PMA. PMA acts as a diacylglycerol analog to activate protein kinase C (PKC) [63,241]. However, it has been shown that PKC isoforms are modulated differently by PMA between cell lines (Jurkat and H9 T-cells) [242]. Concerning HIV-1, it has been established that PKC stimulates NF-κB signaling, thereby inducing transcription. In contrast, activation of HTLV-1 transcription is not mediated by NF-κB but instead displays an involvement of Sp1, p53, and CREB upon TPA-treatment [63,242,243].

Apart from that, PKC triggers reactive oxygen species (ROS) production, thereby causing DNA damage and cell stress [244,245]. As a consequence, PMA also induces apoptosis. Strikingly, the apoptotic cascade seems to be linked to latency reversal [245]. In HTLV-1 infected T-cell lines (HUT 102, FS, 1996, and 1657), PMA can enhance Tax protein expression [117]. PMA treatment is often combined with the Ca^2+^ ionophore ionomycin. Ionomycin is a T-cell activator and stimulates latent viral transcription. Moreover, ionomycin can act synergistically with PMA to activate PKC [246,247]. Since PMA is carcinogenic, it has not been pursued as an LRA for treatment. Nevertheless, it is frequently used as a positive control for activation of latent viral transcription in in vitro experiments [111,248].

### 4.3. Sirtuin Inhibitors

The non-zinc-dependent but rather NAD^+^-dependent class III of HDACs has been briefly introduced in Section 2.1.1. This class of HDACs is designated as sirtuins (SIRTs), consisting of isoforms SIRT 1-7. These isoforms are widely expressed in mammals and further divided according to their subcellular localization. SIRT 1, 6, and 7 are found in the nucleus, SIRT 2 in the cytoplasm, and SIRT 3, 4, and 5 in the mitochondrion [92,136,249]. Potential substrates of SIRTs are histone proteins, but also non-histone proteins, such as NF-κB and p53. Consequently, SIRTs are involved in different physiological processes, ranging from metabolism to apoptosis. Besides, they are implicated in various diseases [92,249,250].

It has been observed that SIRT 1 is overexpressed in ATL patients and acts as an antiapoptotic molecule in malignant cells. This is supported by the finding that the SIRT 1/2 inhibitor sirtinol caused growth and cell cycle arrest and apoptosis in PBMCs from ATL patients and HTLV-1 positive cell lines (S1T, MT-2). Furthermore, sirtinol blocked NF-κB activity by inhibiting its translocation into the nucleus [249,250,251].

Interestingly, SIRT 1 can suppress Tax activation of the HTLV-1 LTR. However, SIRT 1 inhibitors, such as sirtinol and Ex527, can reverse this effect (Table 3). Administration of these agents to HTLV-1 positive cell lines (C8166, MT-2, and MT-4) enhanced the LTR-driven production of *Tax* mRNA [252]. Novel small-molecule inhibitors of SIRT 1, such as NCO-01 and NCO-04, which have induced apoptosis in cells from ATL patients, might be even more effective at inducing Tax expression [253]. Therefore, inhibition of SIRT 1 may also pose an option to reverse HTLV-1 latency.

### 4.4. Extracellular Factors and Circumstances

Once HTLV-1 infected PBMCs are introduced into culture, they display strong spontaneous transcriptional reactivation [24,256]. Of late, it has been shown that p38 mitogen-activated protein kinases (p38 MAPK) are implicated in this phenomenon, as they are sensors of extracellular stress. MAPKs induce the expression of immediate-early genes (IEGs). Interestingly, the HTLV-1 5′ LTR displays similarities to the promoters of IEGs [24,254,257]. The relevance of MAPKs in the reactivation of HTLV-1 is corroborated by the observation that reactive oxygen species, causing oxidative stress, induce Tax expression in MT-1-GFP^+^ reporter cells [110] (Table 3). There are further ramifications in the spontaneous reactivation of HTLV-1, oxygen availability being a major factor. Physiological hypoxia, which represents 1–2% oxygen, can be found in anatomical departments, such as parts of the central nervous system and stem cell niches [258]. These oxygen levels enhance transcription from the HTLV-1 5′ LTR in HTLV-1 infected PBMCs. This enhancement is likely due to hypoxia being a trigger for p38 MAPK. Beyond that, hypoxia shifts the glucose metabolism towards glycolysis [24,254,255]. Remarkably, glucose metabolism was demonstrated to be crucial for HTLV-1 reactivation. Inhibition of glycolysis or the mitochondrial electron transport after overnight culture decreased *Tax* transcription [24,255]. Further investigation of the circumstances of spontaneous transcriptional reactivation could reveal additional targets for reversing HTLV-1 latency.

## 5. Caveats of the Kick and Kill Approach and Open Questions

Despite promising results in cell culture experiments and in ex vivo analyses of fresh peripheral blood mononuclear cells from HTLV-1-infected patients, the first clinical trials using HDACi did not lead to a sustained reduction of the proviral load, nor could the patients be cured. However, until now, most trials in HTLV-1-infected patients have been focusing on valproate, which is known to be far less potent than the newer classes of HDACi that have been developed in the meantime [100]. Thus, future work should address the impact of novel classes of HDACi and should also consider modifying viral transcription more specifically by modulating P-TEFb. It may also be feasible to combine HDACi and P-TEFb modulators, a strategy that has already been shown to have superior effects on viral reactivation in cell culture models of HIV compared to individual treatment regimens [198,230]. Although an increase in viral transcription may result in enhanced immunogenicity of HTLV-1, this approach also has limitations:

- Yet, it is unclear whether increased viral transcription also results under any conditions in enhanced viral protein expression and antigen presentation. For HIV, several studies have described post-transcriptional blocks, for HTLV-1, this is unknown [131,132].
- The treatment of HTLV-1-infected patients with HDACi or related compounds does not specifically target virus-infected cells. Rather, other cells like CTLs themselves may also be affected by the treatment and may be functionally impaired. Thus, individual compounds have to be monitored more closely.
- Enhanced viral transcription may result in increased viral replication and worsening of the phenotype if the CTL response does not work properly. Since viral reactivation also poses the risk that a yet repressed but harmful HTLV-1-infected T-cell clone proliferates upon treatment, close monitoring of patients would be required.
- It is unclear whether the combination of antiretroviral therapy with different compounds used for the Kick and Kill strategy could improve clinical parameters comparable to the approaches used in HIV reactivation.
- The outcome of viral reactivation may differ depending on the viral integration sites in the human genome.
- Viral reservoirs are largely unknown for HTLV-1. Most studies performed thus far monitored the impact of LRAs on peripheral blood mononuclear cells. However, whether and how LRAs may impact viral gene expression in other cell types and tissues and how accessible these viral reservoirs are, is not understood.
- The impact of LRAs on HBZ expression and HBZ-specific CTLs remains to be elucidated in more detail.

Summing up, more work is needed to gain novel insights into host factors and chemical compounds required for manipulating HTLV-1 gene expression, and thus, the immunogenicity of HTLV-1. This holds important implications for the Kick and Kill strategy to relieve the disease burden of ATLL and HAM/TSP.

## Figures and Tables

**Table 1:** Different HDACi that reverse retroviral latency.

HDACi	HDAC Selectivity	Use in Latency Reversal or Retroviral Infection		Result	Ref.
		HIV-1	HTLV-1 or Related Viruses		
**Hydroxamates**					
Trichostatin A (TSA)	pan-HDACi	🗸	HTLV-1 pos. cell lines (MT-2, SLB-1), PBMCs from BLV-infected sheep	enhancement of global histone acetylation increase of viral transcription and expression	[69,70,107,108,109]
Vorinostat (Suberoylanilide hydroxamic acid, SAHA, Zolinza)	pan-HDACi	🗸	MT-1-GFP^+^ reporter cells	induction of Tax expression	[105,108,110]
Belinostat (PXD-101, Beleodaq)	pan-HDACi	🗸	X	induction of HIV-1 viral production	[84,105,111]
Panobinostat (LBH589, Farydak)	pan-HDACi	🗸	MT-1-GFP^+^ reporter cells	induction of Tax expression greater potency than other Hydroxamates	[105,110,111]
**Benzamides**					
Entinostat (MS-275)	class I	🗸	HTLV-1 pos. cell lines (MT-1, -2, -4)	growth inhibition of HTLV-1 pos. cell lines	[95,112,113]
Chidamide (HBI-8000, Epidaza)	class I, class IIb	🗸	ATLL-derived cells lines	proapoptotic in ATLL-derived cells lines decrease in cell-associated HIV-1 DNA	[84,102,114,115]
**Short-chain fatty acids**					
Butyrates	class I, class IIa	🗸	HTLV-1 pos. cell line(SLB-1, HUT 102)	enhancement of histone acetylation at HTLV-1 promoter increase of viral transcription and Tax protein expression	[70,108,116,117]
Valproate (Valproic acid)	class I, class IIa	🗸	cell and animal models (BLV, STLV), HAM/TSP patients	increase in transcription from viral promoter raise of proviral load	[57,66,101,118,119,120]
**Cyclic peptides**					
Romidepsin (FK228, Depsipeptide, Istodax)	class I	🗸	HTLV-1 pos. cell lines (HUT 102, MT-2)	increase in histone acetylation induction of Tax protein expression	[84,121,122]

Abbreviations: HDACi, histone deacetylase inhibitor; HDAC, histone deacetylase; HIV-1, human immunodeficiency virus type 1; HTLV-1, human T-cell leukemia virus type 1; PBMC, peripheral blood mononuclear cells; BLV, bovine leukemia virus; ATLL, adult T-cell leukemia/lymphoma; STLV, simian T-cell leukemia virus.

**Table 2:** Different P-TEFb-modulating agents that reverse retroviral latency.

	Mode of Action	Use in Latency Reversal or Retroviral Infection		Result	Ref.
		HIV-1	HTLV		
**LMW-complex**					
JQ1	BRD4 inhibitor	HIV-1 pos. cell lines (A2, A72, J-Lat clone 10.6)	🗸	induction of cyclinT1 expression increase of transcription from HIV-1 promoter suppression of proliferation, blocking of cell cycle progression, and induction of apoptosis in Tax-expressing cells induction of Tax expression in MT-1-GFP^+^ reporter cells	[72,110,203,204,206]
PEP005	BRD4 inhibitor	cell line (ART patients)	X	increase of transcription from HIV-1 promoter synergistically with JQ1	[205]
iBET151 (GSK1210151A)	BRD4 inhibitor	HIV-1 pos. cell lines	X	induction of HIV-1 gene transcription	[72,207]
Birabresib (OTX-015)	BRD4 inhibitor	AML and ALL cell lines, cell line (ART patients)	X	growth inhibition of AML and ALL cell lines greater potency than JQ1	[208,209]
UMB-136	BRD4 inhibitor	HIV pos. cell lines harboring latent proviruses	X	induction of HIV-1 viral production	[210]
**HMW-complex**					
HMBA	PI3K/Akt pathway activation/	chronically infected cells (U1 ACH-2, and JΔK)	X	induction of viral production	[71,169,211,212]
Vorinostat (SAHA)	CDK9 T-loop phosphorylation	resting CD4^+^ T cells (aviremic patients)	X	increase of HIV RNA expression in vivo movement of CycT1 and CDK9 from higher (7SK snRNP) to lower (free P-TEFb) glycerol fractions	[130,169,211,213,214]
Disulfiram	PI3K/Akt pathway activation/	cell line (HAART patients) dose-escalation study	X	increase of cell-associated unspliced HIV RNA	[215,216,217]
Trichostatin (TSA)		reporter in 293T cells	X	30-fold induced HEXIM1 promotor activity	[169,218]
5-Azacytidine (AzaC)	analog of cytidine	HeLa cells 293T cells	🗸	release of P-TEFb from the 7SK snRNP stimulation of luciferase activity by 5-fold, similar to levels achieved by HMBA	[185,47]

Abbreviations: HIV-1, human immunodeficiency virus type 1; HTLV-1, human T-cell leukemia virus type 1; LMW, low molecular weight; BRD4, bromodomain-containing protein 4; ART, antiretroviral therapy; AML, acute myeloid leukemia; ALL, acute lymphatolastic leukemia; HMBA, hexamethylene bisacetamide; PI3K, phosphatidyl-inositol 3-kinase; CDK9, cyclin-dependent kinase 9; 7SK snRNP, 7SK small nuclear ribonucleoprotein; P-TEFb, positive transcription elongation factor b; HAART, highly active antiretroviral therapy; HEXIM1, hexamethylene bisacetamide inducible protein 1.

**Table 3:** Diverse stimuli that are involved in HTLV-1 latency reversal and transcriptional activation.

Stimulus	Mode of Action	Use in HTLV-1 Infection	Result	Ref.
**Mitogens**				
Phytohemagglutinin-P (PHA)	T-cell activation	HTLV-1 pos. cell line (FS)	induction of Tax protein expression	[117]
Phorbol 12-myristate 13-acetate (PMA)	PKC activation	HTLV-1 pos. cell lines (HUT 102, FS, 1996, 1657)	induction of Tax protein expression	[117]
**SIRT inhibitors**				
Sirtinol	SIRT 1 inhibition	PBMCs from ATLL patients, HTLV-1 pos. cell lines	growth and cell cycle inhibition, apoptosis increase of *Tax* mRNA	[251,252]
Ex527	SIRT 1 inhibition	HTLV-1 pos. cell line (C8166)	increase of *Tax* mRNA	[252]
**Extracellular factors and circumstances**				
Oxidative stress	p38 MAPK- activation	HTLV-1 pos. cell line (MT-1-GFP^+^ reporter cells)	enhancement of Tax expression	[110]
Physiological hypoxia	p38 MAPK- activation	PBMCs from HTLV-1 infected patients	increase in HTLV-1 5′ LTR transcription	[254,255]
Glucose metabolism	link to Tax protein pos. feedback loop	PBMCs from HTLV-1 infected patients	imperative for HTLV-1 5′ LTR transcription	[24,254,255]

Abbreviations: HTLV-1, human T-cell leukemia virus type 1; PBMC, peripheral blood mononuclear cells; PKC, protein kinase C; SIRT, sirtuin; ATLL, Adult T-cell leukemia/lymphoma; MAPK, mitogen-activated protein kinases.
